# Combining SNP discovery from next-generation sequencing data with bulked segregant analysis (BSA) to fine-map genes in polyploid wheat

**DOI:** 10.1186/1471-2229-12-14

**Published:** 2012-01-26

**Authors:** Martin Trick, Nikolai Maria Adamski, Sarah G Mugford, Cong-Cong Jiang, Melanie Febrer, Cristobal Uauy

**Affiliations:** 1 John Innes Centre, Norwich Research Park, Colney, Norwich NR4 7UH, UK; 2 The Genome Analysis Centre, Norwich Research Park, Colney, Norwich NR4 7UH, UK; 3 National Institute of Agricultural Botany, Huntingdon Road, Cambridge CB3 0LE, UK

## Abstract

**Background:**

Next generation sequencing (NGS) technologies are providing new ways to accelerate fine-mapping and gene isolation in many species. To date, the majority of these efforts have focused on diploid organisms with readily available whole genome sequence information. In this study, as a proof of concept, we tested the use of NGS for SNP discovery in tetraploid wheat lines differing for the previously cloned grain protein content (GPC) gene *GPC-B1*. Bulked segregant analysis (BSA) was used to define a subset of putative SNPs within the candidate gene region, which were then used to fine-map *GPC-B1*.

**Results:**

We used Illumina paired end technology to sequence mRNA (RNAseq) from near isogenic lines differing across a ~30-cM interval including the *GPC-B1 *locus. After discriminating for SNPs between the two homoeologous wheat genomes and additional quality filtering, we identified inter-varietal SNPs in wheat unigenes between the parental lines. The relative frequency of these SNPs was examined by RNAseq in two bulked samples made up of homozygous recombinant lines differing for their GPC phenotype. SNPs that were enriched at least 3-fold in the corresponding pool (6.5% of all SNPs) were further evaluated. Marker assays were designed for a subset of the enriched SNPs and mapped using DNA from individuals of each bulk. Thirty nine new SNP markers, corresponding to 67% of the validated SNPs, mapped across a 12.2-cM interval including *GPC-B1*. This translated to 1 SNP marker per 0.31 cM defining the *GPC-B1 *gene to within 13-18 genes in syntenic cereal genomes and to a 0.4 cM interval in wheat.

**Conclusions:**

This study exemplifies the use of RNAseq for SNP discovery in polyploid species and supports the use of BSA as an effective way to target SNPs to specific genetic intervals to fine-map genes in unsequenced genomes.

## Background

Wheat is a major food staple providing approximately 20% of the calories and protein consumed by humankind [1]. The two major cultivated wheat species are tetraploid durum wheat (*Triticum turgidum *ssp. *durum*), which is normally used for pasta-making, and hexaploid bread wheat (*T. aestivum*), which is used for bread and biscuit-making. Both of these polyploid species have large genome sizes (5.3 Gb per haploid genome) [2], a high proportion of repetitive elements (~85-90%) [3], low gene density, and lack an assembled whole genome sequence. This makes the identification of genes through both forward and reverse genetic approaches a time-consuming and challenging task.

To identify genes in wheat, traditional positional cloning projects are often undertaken, although they are low-throughput and extend for many years. The initial gene discovery phase involves a genome-wide survey and, in the case of quantitative traits, is coupled to a subsequent validation of the most relevant candidate regions using near isogenic lines [4,5]. The ensuing steps towards gene isolation include fine-mapping by increasing marker density across the target region using the syntenic relationships with the sequenced cereal genomes (*Brachypodium distachyon, Oryza sativa*, and *Sorghum bicolor*) [6-10]. The final steps involve development of physical maps [11,12], in many cases using dedicated BAC libraries [13], and candidate gene validation [14,15]. This strategy has been used successfully to clone several wheat genes with major effects as well as QTL (reviewed in [16]). However, these examples stand out as isolated cases. New approaches are required for step-changes in the speed and effectiveness in which genes are mapped and cloned in polyploid wheat.

Next-generation sequencing (NGS) technologies are providing new ways to accelerate the genetic analysis of traits. One application of NGS is to use whole genome re-sequencing to aid in the fine-mapping and identification of causal polymorphisms. Several strategies have recently been published in the model plant *Arabidopsis thaliana *and have been collectively termed as 'NGS-enabled genetics' [17]. The SHOREmap method [18] uses a single NGS reaction to perform genome-wide re-sequencing of a large bulk of mutant F_2 _individuals that allows the identification of the causative single nucleotide polymorphism (SNP) in the gene of interest. Austin and co-workers [19] propose a modified version of this approach (termed 'next-generation mapping') which requires much smaller F_2 _populations than SHOREmap and uses a statistic to qualitatively characterize SNP frequencies. Another approach, termed 'fast forward genetics', combines bulked segregant techniques with genome capture technology to identify candidate genes [20]. These three methods highlight the power of NGS and how different approaches can be combined to accelerate fine-mapping and cloning of genes in a diploid model organism, provided the complete genome sequence and a detailed SNP catalogue are available.

The ability to accelerate fine-mapping and cloning of genes in polyploid wheat would have a huge impact on our ability to understand the fundamental biology of this important crop species and would enable wheat breeders to directly access the genetic variation in genes encoding important agronomic traits. The recent development of methods for SNP detection in the transcriptome of non-sequenced polyploid species [21-23] provides an opportunity to use 'NGS-enabled genetics' [17] for this purpose. To address this, we evaluated the use of NGS for both SNP discovery and bulked segregant analysis (BSA) [24] to fine-map a previously cloned gene in polyploid wheat (*GPC-B1*) [14]. Using NGS on mRNA samples (RNAseq), we identified over 3,500 putative SNPs between parental lines and examined their frequency in the two bulked samples of recombinant lines with contrasting phenotype. We mapped 39 new SNPs across the 12.2-cM interval (1 SNP every 0.31 cM) and fine-mapped *GPC-B1 *to approximately 0.4-cM and within 13-18 genes in the syntenic cereal genomes.

## Results

### Assignment of Illumina reads to reference wheat unigenes

Two near-isogenic lines segregating for the *GPC-B1 *grain protein content locus were selected to identify SNPs across this chromosomal region. The first line was tetraploid durum wheat Langdon (LDN), which carries a non-functional allele at *GPC-B1*. The second line was tetraploid recombinant substitution line 65 (RSL65), which is derived from a cross between LDN and a wild emmer (*Triticum turgidum *ssp. *dicoccoides*) chromosome 6B substitution line [LDN (DIC6B)][4]. RSL65 carries a chromosome 6B segment of wild emmer of at least 30 cM that includes a functional *GPC-B1 *allele, but should be isogenic to LDN outside this interval. To reduce variation from growing conditions, parental lines LDN and RSL65 were grown together in the same pot. Total RNA was extracted from leaves at 5th leaf stage and the non-normalized samples were prepared for mRNA-seq on the Illumina GAIIx (120 cycles, paired end (PE), one lane per parent). A flow-chart of the complete process is available in Additional File 1: Figure S1.

A total of 25.6 and 31.7 million paired end 120 base reads were obtained for LDN and RSL65, respectively, after excluding low quality reads and trimming for adaptor contamination and low quality regions. There is currently no reference genome sequence available for wheat, therefore individual reads from each parent were aligned to the NCBI wheat transcriptome [25], which comprises 40,349 unigene sequences, totaling 31,671,110 bases. Almost 50% of LDN raw reads (25.5 million) mapped to the reference unigenes with 78.6% of these mapping in pairs. For RSL65, 47% of raw reads (29.9 million) mapped to the NCBI Unigene reference, with 67.2% of them mapping in pairs. Taken together, of the total 114.6 million Illumina reads produced, 48.3% mapped to the NCBI Unigene reference.

**Figure 1:**
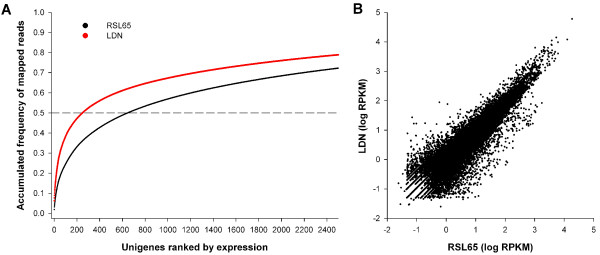
**Accumulated frequency of mapped reads and correlation in transcript levels between LDN and RSL65**. **A) **Accumulated frequency of the percentage of mapped reads in LDN (red) and RSL65 (black) to unigenes ranked from highest to lowest expression level (expressed as RPKM, reads per kilobase per million mapped reads). The broken line corresponds to 50% of all mapped reads. **B) **Comparison of unigene transcript levels (log RPKM) between parental lines. Each unigene is represented by a small circle.

RNA was not normalized in this study as the objectives were both SNP discovery and subsequent identification of allele frequencies between bulked samples. To examine the consequences of this approach on the sequencing of highly expressed transcripts, the relative expression level of unigenes was estimated by calculating the transcript abundance expressed as reads per kilobase per million mapped reads (RPKM) [26]. Unigenes were ranked according to their RPKM (from highest to lowest) and the accumulated frequency of mapped reads calculated (Figure 1A). For the LDN dataset, 14.8% of all mapped reads correspond to the 10 most expressed unigenes (including genes related to photosynthesis and a repetitive element) and half of all mapped reads correspond to the 248 most highly expressed unigenes. In RSL65, these values are slightly greater with 50% of mapped reads aligning to the 647 most highly expressed unigenes. The slope of the curve in Figure 1A decreases after the 50% value in both parental lines. This suggests that the lack of normalization results in the extreme over-representation of a few transcripts (50% of reads map to 0.98% or 2.47% of all unigenes with mapped reads in LDN and RSL65, respectively).

Despite this and the incomplete reference used, there was sufficient sequencing depth in a single Illumina lane to align reads (with at least 8-fold coverage) to 24,083 and 25,080 unigenes in LDN and RSL65, respectively. This represents approximately 60% of the unigene reference set that was present in the parental data. Comparison of unigene transcript levels (expressed as RPKM) between parental lines showed a high correlation (R^2 ^= 0.85, Figure 1B) suggesting that the approach followed should be efficient for both SNP discovery and the bulked segregant analysis later described.

### SNP discovery in near-isogenic lines of tetraploid wheat

In polyploids, SNP discovery is confounded by the presence of two types of SNPs. The first corresponds to polymorphisms between homoeologous genomes that occur within homozygous individuals. These SNPs are commonly found in tetraploid (A and B genomes) and hexaploid wheat (A, B and D genomes) as the homoeologous genomes share sequence identities of ~96-98% [27]. These SNPs are referred to as inter-homoeologue polymorphisms (IHP) [21]. The second type of polymorphism corresponds to varietal SNPs between individuals, representing what is traditionally referred to as allelic variation. This type of SNP is much less frequent with modern wheat varieties being ~99.9% identical across corresponding orthologous loci. Since the transcript assemblies within the reference wheat unigenes represent a consensus sequence derived from the co-assembled A, B and D genomes (i.e. without ambiguity codes, just like the singletons), the two types of SNPs cannot be distinguished by simple SNP discovery pipelines. Therefore, SNPs were detected and scored based on methods previously developed for the polyploid oilseed rape *Brassica napus *[21,28].

**Figure 2:**
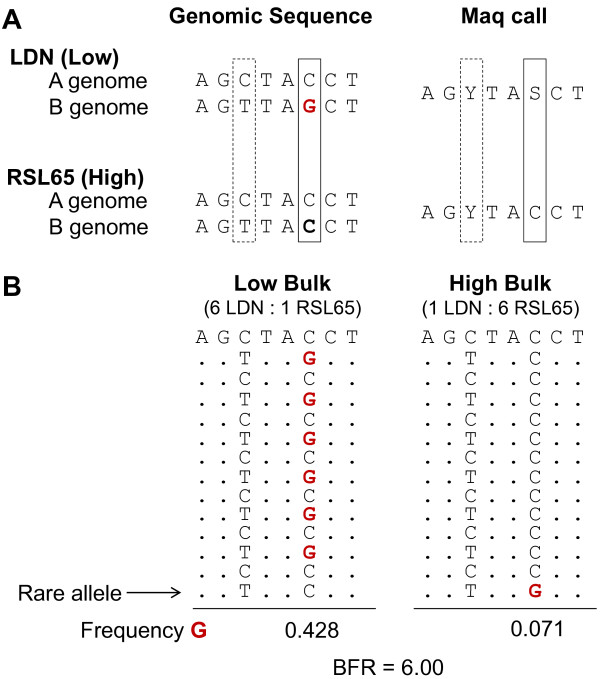
**Schematic representation of SNP identification and BSA in polyploid genomes**. **A) **Schematic representation of a homoeologous SNP between the A and B genome (C/T, broken line rectangle) and of an inter-varietal SNP (or hemi-SNP) between LDN and RSL65 (C/G, full line rectangle). The homoeologous SNP generates the same Maq ambiguity code in both parents (C/T = Y), whereas the hemi-SNP generates a unique ambiguity code (S) in the parental line with the informative base (G in bold red). Figure adapted from [21]. **B**) Schematic representation of the composition of alleles for a SNP that is closely linked to *GPC-B1 *in the low and high bulks. The reference A genome is indicated at the top with additional reads aligning below. The homoeologous SNP (C/T) and the hemi-SNP (C/G) are shown. The ratio between LDN and RSL65 plants in each bulk is shown (LDN:RSL65), as is the calculated BFR of the informative base G.

In this approach, it is expected that reads originating from homoeologous genes (A and B genomes in tetraploid wheat) will be mapped to the same unigene reference. Maq (default parameters)[29] was used to call SNPs with respect to the reference for each parental line separately, thus generating two SNP sets. This was followed by a second step in which a custom Perl script was used to derive the symmetric difference of the two parental SNP sets. Polymorphisms between homoeologous genomes should generate the same ambiguity code in parental lines and should be common to both SNP sets (Figure 2A, C/T generates a Y code). On the other hand, varietal SNPs between LDN and RSL65 should generate an ambiguity code for only one parent and therefore be unique to that corresponding SNP set (Figure 2A, full-line boxed SNP). Such varietal SNPs at the same locus were termed "hemi-SNP" by Trick et al. [21]. In cases when only one genome is expressed or for single copy genes, SNPs could be identified as traditional "simple-SNPs" with each parent represented by a single base.

The implementation of this two-step pipeline identified 3,963 putative varietal SNPs across 2,520 unigenes between LDN and RSL65. The vast majority of the SNPs discovered (80.8%) were of the hemi-SNP type, manifested as ambiguity codes and signifying co-expression of homoeologous genes, which have a particular nucleotide polymorphism in one parent only. In these circumstances, the hemi-SNP then becomes allelic and serves as a genetically tractable marker.

A concern when working with non-sequenced and complex genomes such as those of wheat is the presence of closely related paralogues or pseudogenes that could confound SNP discovery and downstream marker design and mapping. Evidence of paralogues being mapped to the same reference sequence would be revealed as unigenes with high SNP density. Therefore, SNP densities were calculated for the 2,520 unigenes with at least one varietal SNP and their frequency distribution examined. The average SNP density was 1.80 SNPs/kb (± 1.46), and decayed rapidly after 3 SNPs/kb. This value is similar to that calculated empirically (2.2 SNPs/kb) by sequencing 6.8 kb from exons and UTRs of four genes across the *GPC-B1 *region [14]. Based on these results, unigenes with SNP densities higher than 5 SNPs/kb were eliminated to avoid possible paralogues yet still allowing space for some variation. Roughly 13% of putative SNPs were discarded, reducing the number to 3,427 putative SNPs across 2,427 unigenes (1.80 SNPs/kb).

### SNP discovery using modified alignment and SNP calling criteria

Visual examination of several SNPs revealed that Maq was not able to properly call SNPs occurring close to homoeologous SNPs or within a 10-bp sliding window. This meant that some IHPs were being called as varietal hemi-SNPs because of their close proximity. Therefore, the default Maq parameters were relaxed by setting the maximum summed quality score of mismatched bases to 120 (default 70) at each step in the workflow (referred to as Maq-120 hereafter). This allowed SNP haplotypes occurring within the 120 base reads to align with higher mapping quality and reduce these errors.

The Maq-120 analysis produced some improvements in terms of total reads mapped to the unigene reference (55.2% across both parental lines compared to 48.3% in Maq-default), although the percentage of reads that mapped in pairs remained constant (Table 1). These reads aligned with at least 8-fold coverage to 25,262 and 26,180 unigenes in LDN and RSL65, respectively (approximately 63% of the NCBI Unigene set). A total of 6,035 putative SNPs were identified across 3,195 unigenes (2.41 SNPs/kb), which was a considerable increase compared to the Maq default pipeline. Again, hemi-SNPs were most predominant with over 89% of SNPs being assigned to this type. After filtering for unigenes with > 5 SNPs/kb, roughly one quarter of putative SNPs were discarded (1,605 SNPs across 226 unigenes). The final outputs of the Maq-120 analysis were 4,430 putative SNPs across 2,969 unigenes (1.90 SNPs/kb).

**Table 1:** Sequencing statistics and mapping results for parental lines using Maq-default and Maq-120 analysis

			Maq-default analysis				Maq-120 analysis			
**Library**	**PE reads**	**Raw reads**	**raw reads mapping**	**%**	**reads in pairs**	**% ^1^**	**raw reads mapping**	**%**	**reads in pairs**	**% ^1^**

LDN	25,602,724	51,205,448	25,496,798	49.8%	20,052,438	78.6%	29,290,176	57.2%	22,863,235	78.1%
RSL65	31,721,926	63,443,852	29,892,394	47.1%	20,079,735	67.2%	34,014,128	53.6%	22,244,586	65.4%
Total	57,324,650	114,649,300	55,389,192	48.3%	40,132,173	72.5%	63,304,304	55.2%	45,107,821	71.3%

^1 ^= (reads in pairs/raw reads mapping)*100

### Identification of putative linked SNPs via bulked segregant analysis

Twenty eight homozygous lines with recombination events across a ~12-cM interval (*Xwms508 *-*Xwms193*) [5] were used to assemble bulks for contrasting phenotype (grain protein concentration). The lines were first characterized for three markers spanning the 250-kb region adjacent to *GPC-B1 *to confirm their genotype. All lines produced the expected results, except RSL135, which was heterozygous for all markers and was therefore excluded from downstream work. Equal amounts of total RNA from the 14 individuals previously classified as having high protein content were mixed to produce the high protein RNA bulk, whereas 15 recombinant lines were used for the low protein bulk (for detailed description of these lines see 'Materials and Methods'). For each bulk, two libraries with slightly different average insert size (250-bp and 400-bp) were constructed and sequenced in individual Illumina lanes (80 cycles, paired end).

The quality control assessment of reads was similar to the parental lines. For both the Maq-default and the Maq-120 analysis, a higher percentage of reads mapping to the reference set was obtained in the 400-bp libraries compared to the 250-bp libraries (~25% more reads mapping in the former), although a smaller number of reads aligned in pairs (Table 2). Combining both libraries, a total of 53.4 and 61.2 million paired end reads were produced for the High and Low bulk, respectively. Again, the Maq-120 analysis was more successful in aligning a higher percentage of reads to the reference (47.6%) compared to the Maq-default analysis (41.8%). These values are similar to those obtained in the parental lines when comparing only the equivalent 400-bp libraries.

**Table 2:** Sequencing statistics and mapping results of bulked samples using Maq-default and Maq-120 analysis

				Maq-default analysis				Maq-120 analysis			
	**Library**	**PE reads**	**Raw reads**	**raw reads mapping**	**%**	**reads in pairs**	**% ^1^**	**raw reads mapping**	**%**	**reads in pairs**	**% ^1^**

High Bulk	400-bp	21,501,822	43,003,644	20,481,156	47.6%	17,044,066	83.2%	22,978,578	53.4%	19,080,072	83.0%
	250-bp	31,917,705	63,835,410	22,992,792	36.0%	20,270,532	88.2%	26,489,852	41.5%	22,917,204	86.5%
	Total	53,419,527	106,839,054	43,473,948	40.7%	37,314,598	85.8%	49,468,430	46.3%	41,997,276	84.9%
Low Bulk	400-bp	28,215,779	56,431,558	27,059,022	48.0%	22,074,113	81.6%	30,353,820	53.8%	24,719,488	81.4%
	250-bp	32,981,686	65,963,372	25,389,354	38.5%	22,597,308	89.0%	29,236,028	44.3%	25,539,200	87.4%
	Total	61,197,465	122,394,930	52,448,376	42.9%	44,671,421	85.2%	59,589,848	48.7%	50,258,688	84.3%

^1 ^= (reads in pairs/raw reads mapping)*100

The objective of sequencing RNA from bulked samples was to compare the allelic frequencies for the parental SNPs between the two bulks. In a diploid organism, a SNP coinciding with the gene underlying the trait of interest should be revealed by informative base frequencies tending to either 1.0 or to 0.0 in the two bulks, depending on the parental origin. However, in polyploid species this upper limit of 1.0 is lower. For example, for a hemi-SNP identified in LDN (C/G) with respect to RLS65 (C), the signal from the informative base (G) is partially masked by the homoeologous (non-informative) base (C) (Figure 2B). Therefore, the upper limit frequency of the informative base would tend to 0.5 in tetraploid species, assuming quantitative co-expression of the two homoeologous transcripts. This value would change when relative transcript levels depart from parity. For each bulk, the frequency of the informative base was calculated at each SNP position and then the ratio between the bulks was determined for each SNP. This ratio between bulks was termed bulk frequency ratio (BFR). For hemi-SNPs with an informative base derived from LDN, the BFRs were determined by dividing the frequency in the low bulk by the frequency in the high bulk. For hemi-SNPs derived from RSL65, these values were reversed. Thus, the BFR provides a relative measure of the enrichment of the corresponding parental allele in the appropriate bulk (LDN for the low bulk and RSL65 for the high bulk).

The approach focused exclusively on the putative SNPs previously identified between LDN and RSL65 in the initial experiment and did not seek to identify SNPs in the complex bulk RNA mixtures. Of the 3,427 SNPs (2,427 unigenes) identified in the Maq-default analysis, 1,619 SNPs were recovered and had sequence coverage of at least 8-fold in both bulks (Additional File 2: Table S1). In this analysis, only one SNP per unigene was used to estimate the BFR. Therefore, the percentage of SNPs recovered was only 47.2%, even though this represents 66.7% of the unigenes. The highest BFR identified was the simple SNP T115G in unigene Ta#S16259088, which had a 28.5-fold enrichment. The LDN allele was present in only 3.4% of the high bulk sequences, whereas 96.5% of the low bulk reads carried this allele. These values are consistent with the expected frequencies of a tightly linked simple SNP. This unigene was homologous to *Brachypodium *Bradi3g03340, rice LOC_Os02g04500, and sorghum Sb04g003030, which are all in the corresponding collinear regions to wheat chromosome arm 6BS, where *GPC-B1 *maps.

**Figure 3:**
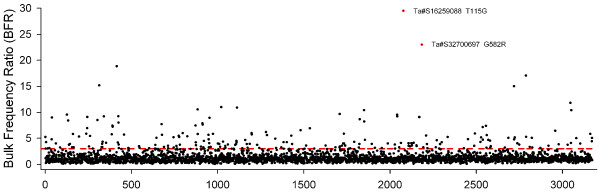
**BFR for putative SNPs identified in Maq-120 analysis**. BFR of 3,172 SNPs with at least 8-fold coverage in the high and low bulk according to Maq-120 analysis. SNPs are plotted along the X-axis according to the unigene rank based on the RPKM expression values. The broken red line indicates the threshold value 3.00. The two SNPs with the highest BFR (Ta#S16259088 and Ta#S32700697) are represented by red dots and are labeled.

Allele frequencies of the Maq-120 putative SNPs (4,430 SNPs across 2,969 unigenes) were also examined in the bulks. In this new pipeline, the frequencies and ratios of multiple SNPs within a single unigene were estimated independently and thus all SNPs were considered. Over 71% of SNPs (3,172) had at least 8-fold coverage and could be detected in both bulks (Figure 3, Additional File 3). Again, the putative SNP with the highest BFR was T115G in unigene Ta#S16259088, although the exact values changed slightly with the Maq-120 pipeline (29.5-fold enrichment). The putative SNP with the second highest BFR was a RSL65 hemi-SNP G582R in Ta#S32700697. The informative base (A as R = G/A) was present in only 1.7% of the low bulk reads, whereas it was found in 39.7% of the reads from the high bulk (23-fold enrichment). These values are consistent with the expectations of a tightly linked hemi-SNP. Again, this unigene has homology to cereal genes that map within the syntenic regions to wheat chromosome arm 6BS (Bradi3g03530, LOC_Os02g04660, Sb04g003160).

### SNP validation

The distribution of the BFRs across both analyses was used to determine which putative SNPs to validate and map (Table 3). Approximately 85% of SNPs had a BFR lower than 2.0, suggesting that both LDN and RSL65 alleles were expressed at relatively similar levels in both bulks. As the BFR threshold increased, the number of SNPs decreased in comparative terms in both analyses. The objective was to use a threshold that was sufficiently low to examine the sensitivity of this approach, while maintaining the number of SNPs to be validated within a manageable number. Therefore, a BFR of ≥ 3.0 was empirically determined as the threshold resulting in a total of 270 SNPs (41 SNPs were common between both analyses) across 253 unigenes.

**Table 3:** Number and percentage of SNPs identified at different BFRs using Maq-default and Maq-120 analyses

BFR	Maq-default		Maq-120	
	
	SNPs	%	SNPs	%
≥ 2.0	225	13.9%	464	14.6%
≥ 3.0	99	6.1%	212	6.7%
≥ 4.0	51	3.2%	113	3.6%
≥ 5.0	33	2.0%	75	2.4%
≥ 6.0	21	1.3%	43	1.4%
≥ 7.0	15	0.9%	36	1.1%
≥ 8.0	10	0.6%	28	0.9%
≥ 9.0	8	0.5%	22	0.7%
≥ 10.0	6	0.4%	12	0.4%

To validate these putative polymorphisms, a single SNP for each unigene was selected for marker development. The 99 putative SNPs from the Maq-default analysis (BFR above 3.0) were first examined. Fifteen SNPs were either located too close to the ends of the available sequence or their assigned unigenes were repetitive as determined by high number of hits to the 5× Chinese Spring wheat genomic sequence (> 100 hits, E-value 1E-50) [30]. Therefore, these 15 SNPs were removed from further analysis. A total of 84 assays were developed of which 82 gave successful amplification in LDN and RSL65. PCR amplicons were visualized through single strand conformation polymorphism (SSCP) and the target polymorphism was detected in 48 unigenes (58.5%), whereas 34 assays (41.5%) were monomorphic. The absence of the putative SNPs in the monomorphic amplicons was confirmed by direct sequencing of PCR products (Additional File 1: Figure S2).

**Figure 4:**
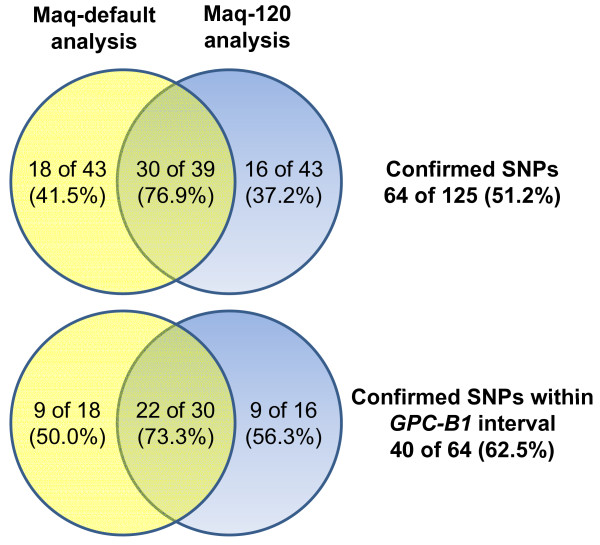
**Comparison of Maq-default and Maq-120 analysis**. Venn diagram of confirmed polymorphic SNPs and those mapping to the *GPC-B1 *interval using the Maq-default (yellow circle), Maq-120 (blue circle) and the combined analysis (intersection of the two circles).

The subsequent Maq-120 analysis yielded a total of 212 putative SNPs with BFRs above 3.0, and 41 SNPs (19.3%) were in common with the Maq-default analysis. Examination of these common putative SNPs showed that of the 39 successful assays previously developed, 30 were polymorphic between LDN and RSL65 (76.9%), whereas only nine were monomorphic. This was a large increase with respect to the average polymorphic rate in the initial Maq-default analysis (58.5% polymorphic) (Figure 4).

To assess if the increase in validation rate was consistent across all the Maq-120 putative SNPs, SNP assays were developed for an additional 43 putative SNPs that were only identified in the Maq-120 analysis. The Maq-120 unique SNPs had a lower validation rate (16 polymorphic, 37.2%) to those of the common SNPs, comparable to the validation rate of the Maq-default unique SNPs (41.5%). In total, 82 putative SNPs were successfully screened from the Maq-120 analysis (39 common and 43 unique) with 46 SNPs being validated as polymorphic (56.1%) and 36 confirmed monomorphic. These results suggest that the Maq-120 analysis is able to identify putative SNPs with a similar conversion rate (56.1%) to that of the Maq-default analysis (58.5%), but that the SNPs common between both analyses produces the putative SNP set with the highest validation percentage (76.9%).

In summary, 64 SNPs (18 unique to Maq-default, 16 unique to Maq-120, 30 common between both methods) from a total of 125 putative SNPs were confirmed (51.2%) as polymorphic in the parental lines.

### Mapping of validated SNPs

The 64 validated SNPs were screened in the individual RSLs that comprised the high and low protein bulks. The SNPs from the Maq-default analysis were screened by SSCP (42 SNPs) and direct sequencing of PCR products (6 SNPs), whereas the confirmed SNPs from the Maq-120 analysis (excluding the common ones with the Maq-default analysis) were screened using KASPar assays (16 SNPs). All confirmed SNPs could be scored in the 29 RSLs and parental lines, except for one SNP (Ta#S32606580), which was very difficult to score unequivocally by SSCP and was therefore removed from further analysis.

**Figure 5:**
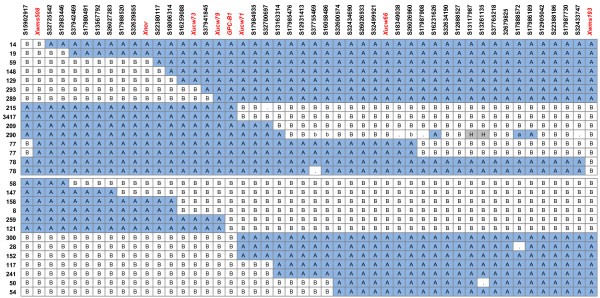
**Graphical genotypes and fine-mapping of *GPC-B1***. Graphical genotypes of RSLs based on SSCP, sequencing and KASPar markers. Markers are scored as A for LDN alleles and B for RSL65 allele. Nomenclature of markers is based on the wheat unigene with the corresponding SNP and previously mapped markers are in red text. A change in colour between adjacent cells indicates a recombination event. Missing values are indicated by dots and lowercase letters indicate data points with low confidence. Sample 290 includes several heterozygous markers (highlighted in grey).

A total of 40 SNPs mapped to the 12.2 cM region encompassing *GPC-B1*, defined by *Xwms508*-*Xwms193 *(Figure 5, Additional File 1: Figure S3). This translates to roughly 63% of validated SNPs mapping to the target region as determined by the RSLs. Twenty-four SNPs could not be mapped immediately linked to this region, although 11 SNPs were linked amongst them. Of the 40 linked SNPs, one mapped just distal to *Xwms508*, whereas 39 SNPs mapped within the 12.2 cM target interval (Figure 5). This equates to an average marker density of one SNP marker per 0.31 cM across this region, with values ranging from one SNP marker per 0.59 cM in the distal end of the map (*Xwms508-GPC-B1*), to one SNP marker every 0.19 cM in the proximal end (*GPC-B1*-*Xwms193*). Almost all recombination events across the region were identified, including additional ones that were previously not resolved. The recombination events not identified correspond to those just flanking the *GPC-B1 *gene on either side, between *Xucw79 *and *Xucw71*. The composition of the 40 SNPs was similar to that of the overall Maq-default and Maq-120 analysis, with a higher number of hemi-SNPs (33) than simple SNPs (7), and a similar proportion coming from each parent (16 LDN and 17 RSL65 SNPs). The SNP marker details are provided in Additional File 4.

Unique orthologues could be determined for 32 of the 40 wheat unigenes in the sequenced *Brachypodium *and rice genomes, whereas 31 unigenes had unique orthologues in sorghum. Based on the established syntenic relationships, 22, 18 and 20 of these orthologues were located on the corresponding syntenic regions in *Brachypodium*, rice and sorghum, respectively. This implies that between 56% (rice) and 69% (*Brachypodium*) of the mapped unigenes are syntenic. These collinear regions encompass between 5.5 and 6.8 Mb and include 665, 989 and 607 genes in *Brachypodium*, rice and sorghum, respectively. The distribution of the orthologous syntenic cereal genes was examined to estimate the number of genes between each SNP in the sequenced genomes. On average, the orthologous syntenic genes were separated by 32, 59 and 32 genes in *Brachypodium*, rice and sorghum, respectively. This average includes markers on adjacent genes to markers separated by at most 103 genes in *Brachypodium *and sorghum and 248 genes in rice. The remaining unigenes have orthologues that map elsewhere in these sequenced grass genomes and there is no apparent pattern except for two unigenes, which are linked in wheat and map closely in *Brachypodium *(Bradi1g67540 and Bradi1g67570), rice (LOC_Os03g15390 and LOC_Os03g15350) and sorghum (Sb01g040520 and Sb01g04530).

The *GPC-B1 *gene was mapped using the phenotypic information previously published for the RSLs [5,14] within a 0.4 cM interval defined by flanking loci Ta#S37941845 and Ta#S17984935 (Table 4, Figure 5). These markers were either completely linked or just one recombination away from the SNPs with the highest BFRs from the Maq-default and Maq-120 analyses (Ta#S16259088 and Ta#S32700697, Figure 3). The homology of these markers to *Brachypodium*, rice and sorghum provides an immediate location for *GPC-B1 *within a region including approximately 18, 16 and 13 genes, respectively. Table 4 shows the characteristics of these markers with their syntenic relationship. These results, together with the distribution of SNP markers across the wheat *GPC-B1 *genetic map, suggest that the enrichment via BSA was very effective at identifying closely spaced markers, enabling the mapping of the gene to a narrow genetic interval.

**Table 4:** Characteristics of the eight SNP markers surrounding the *GPC-B1 *locus

Unigene	Type^1^	Informative base	12-fold^2^	16-fold^2^	Assay	Maq-default	Maq-120	*Brachypodium*	Rice	Sorghum	RPKM^3^	High Bulk	Low Bulk	BFR
Ta#S22380117	H	LDN	YES	YES	SSCP	YES	YES	3g03160	02g04330	04g002930	1.97	0.059	0.226	3.84
Ta#S18006314	S	-	NO	NO	SSCP	YES	NO	3g03270	02g04460	04g002980	51.72	0.255	0.832	3.27
Ta#S16259088	S	-	YES	YES	SSCP	YES	YES	3g03340	02g04500	04g003030	19.92	0.034	0.965	28.46
Ta#S37941845	H	LDN	YES	YES	KASPar	NO	YES	3g03330	02g04490	04g003020	29.92	0.130	0.861	6.60
Ta#S17984935	H	LDN	YES	YES	SSCP	YES	YES	-	02g04650	04g003150	28.85	0.075	0.436	5.84
Ta#S32700697	H	RSL65	YES	YES	KASPar	NO	YES	3g03530	02g04660	04g003160	19.36	0.397	0.017	23.00
Ta#S13163314	H	LDN	YES	YES	KASPar	NO	YES	1g39300	06g30910	10g020020	2.99	0.015	0.230	15.17
Ta#S17985476	H	RSL65	YES	YES	SSCP	YES	YES	3g03480	03g59470	01g004030	23.63	0.156	0.022	7.19

**^1 ^**H = hemi-SNP, S = simple SNP

**^2 ^**SNP present in Maq-120 analysis using 12× and 16× coverage

**^3 ^**RPKM: reads per kilobase per million mapped reads

To further refine this position, we examined the parental SNP data to identify cases where a collinear cereal gene did not have a corresponding wheat unigene, or to assess if any parental SNPs in candidate genes were not identified in the bulks. We analyzed 23 wheat unigenes with homology to genes within the intervals defined in *Brachypodium*, rice and sorghum, but only one had a SNP between LDN and RSL65 (BFR 1.9) and was confirmed to be monomorphic. We next developed an additional nine gene models in wheat for genes that had no corresponding unigene in the reference set used to map the Illumina reads. Again, this search was unsuccessful, as no additional parental SNPs were found based on our mapping criteria. Regardless of these specific results, the ability to examine the data in an iterative manner should be useful for other gene targets.

### Effects of coverage on SNP calling and enrichment in bulks

**Figure 6:**
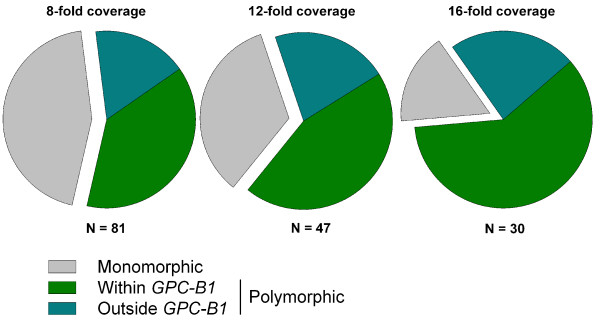
**Effect of coverage on SNP conversion rates and mapping with the *GPC-B1 *interval**. Polymorphic SNPs include those mapping within and outside of *GPC-B1*. N indicates the total number of functional assays considered for each coverage depth. Although the proportion of total SNPs that map within *GPC-B1 *increases, the relative proportion of polymorphic SNPs that map to *GPC-B1 *remains between 67 and 72%. Only SNPs identified using the Maq-120 pipeline were considered for this analysis.

Increasing the depth threshold applied to the whole SNP calling process serves to increase the probability that reads from each homoeologue are sampled. We therefore examined the effect of different minimum depth thresholds (8-fold, 12-fold and 16-fold) on the false positive rate of SNPs identified under the Maq-120 analysis. First, the total number of SNPs with a BFR above 3.0 decreased from 212 using 8-fold coverage, to 121 (12-fold coverage) and 84 putative SNPs using 16-fold coverage. The subset of 82 SNPs from the Maq-120 analysis, which was experimentally tested, was further examined (Figure 6). With 8-fold coverage, 56.8% of predicted SNPs (46 of 81 functional assays) were validated in the parental lines and could be mapped in the recombinants. This value increased considerably with 12-fold and 16-fold coverage, where 66% (31 of 47) and 83% (25 of 30) of the putative SNPs were confirmed to be polymorphic. A similar number of the total putative SNPs was assayed (between 37-40%) under the three coverage scenarios, suggesting that the comparisons are meaningful. In all three cases, 67-72% of the polymorphic SNPs mapped to the *GPC-B1 *target interval. In summary, increasing coverage from 8-fold to 16-fold reduces the total number of putative SNPs identified in the bulks by 60%, increases the validation rate from 57%-83%, but does not affect the percentage of validated SNPs mapping to the target interval (approximately 67-72%).

## Discussion

The use of NGS-enabled forward genetics is revolutionizing the speed and ability to fine-map and clone genes in model diploid organisms [17]. To understand how these approaches could be implemented in a polyploid, non-sequenced species such as wheat, we examined the use of NGS on mRNA transcripts for SNP discovery and combined this with BSA to identify SNPs that were closely linked to the causal gene.

### NGS-enabled genetics in wheat

Our strategy consisted of using near isogenic lines (LDN and RSL65) to focus the SNP discovery on a specific chromosome interval. We hypothesized that the majority of SNPs would map to the ~30 cM interval on chromosome 6B (including both the short and long arms) that was segregating between these lines. The direct analysis of our results in wheat was not possible due to the absence of an assembled wheat genome sequence. Therefore, we conducted this analysis using the most closely related sequenced cereal, *Brachypodium distachyon *[6], acknowledging the fact that only 60-70% of genes have a real syntenic relationship [31-33].

Three *Brachypodium *regions were identified as being over-represented (Additional File 1: Figure S4) including two distinct and separate regions of chromosome 3. This was expected, based on the well established syntenic relationship between Triticeae group 6 chromosomes and *Brachypodium *chromosome 3 [6,34]. The synteny stretches across the short arm of Triticeae group 6 and *Brachypodium *(up to at least Bradi3g09080, 7.2 Mb) and continues in the long arm of wheat 6 and the *Brachypodium *interval starting at approximately 47.4 Mb (Bradi3g45420) and finishing at the end of the chromosome (59.8 Mb; Bradi3g61020) [34]. The short arm syntenic region was enriched across the complete interval, whereas the long arm interval was enriched only in the proximal section. This most likely reflects the fact that RSL65 is not a complete chromosome substitution line and is known to carry a LDN segment in the distal end of chromosome arm 6BL [4].

We also identified a region of *Brachypodium *chromosome 2 (Bradi2g15900-Bradi2g20570) that was unexpectedly enriched for SNPs as its overall syntenic relationship is to wheat chromosome arm 1 L [6,32,34]. Only one of the 66 SNPs identified in this interval (Ta#S13261135, Bradi2g18330) was mapped to the 6BS region and in total only two SNPs (3%) were enriched in the bulks (BFR ≥ 3.0). This stands in contrast to the SNPs mapping to *Brachypodium *chromosome 3, where 351 SNPs were identified and over 13% (46 of 351) were enriched in the bulks. This suggests either the presence of an additional wild emmer chromosome fragment in RSL65, which was previously not identified by molecular markers, or a small insertion of genes into wheat 6B. Overall, the approach was successful at identifying SNPs in the corresponding collinear regions, despite a variable level of background noise across all *Brachypodium *chromosomes. When analyzing the 12-fold and 16-fold coverage data, the background noise decreased, while the three previously identified regions remained consistently above background level (Additional File 1: Figure S4).

We sampled healthy vegetative stage leaves, whose transcriptome should be well represented in the EST collections, yet we were only successful in mapping, at most, 57% of the Illumina reads to the NCBI Unigene reference. This was despite testing several different parameters in Maq, using different library insert sizes, and different thresholds for trimming of reads. In part this is surprising as the Unigene build includes over one million wheat ESTs from different accessions and developmental stages, although the recent sequencing and analysis of wheat chromosome group 1 genes suggests that less than 65% of them are represented in the EST collections [32]. The Maq-default results are consistent with recent studies in *Brassica *[28], which also used a unigene set drawn from 800,000 ESTs as a reference, but with 80 base reads. We further examined our reads by remapping those that were initially unmapped to the NCBI unigenes against a 5× assembly of 454 'Chinese Spring' wheat genomic sequences (M. Bevan, JIC, personal communication). We found that 57% of the unmapped LDN reads and 65% of the unmapped RSL65 reads aligned to this new reference, implying that at least 80-85% of the Illumina reads are in fact expressed wheat sequences. These results also agree with recent 454 RNAseq data from flag leaves 12 days after anthesis [35]. In this study, 1,460 novel transcripts were assembled that had no sequence similarity (BLASTN, E value, 1E-10) to any NCBI wheat EST. These results highlight the incomplete nature of the current NCBI reference wheat Unigene set.

A consequence of this sampling strategy was that we eliminated the possibility of identifying the causal *GPC-B1 *SNP. This gene is expressed only after anthesis [14] and was not detected in the vegetative tissue used for the RNAseq experiments. This was done consciously as our objective was to test the performance of the approach using the information that was available during the original cloning project. The senescence associated expression pattern was only uncovered during the final stages of the positional cloning [36], therefore sampling was done in the most unbiased way possible. We also considered the under-representation of senescence associated transcripts in the NCBI Unigene reference that was recently confirmed in parallel work [35] and, which we predicted, would have resulted in even fewer mapped reads and an overall decrease in the power of the examined approach.

A key feature in any attempt to perform NGS-enabled genetics in wheat is the necessity to reduce the complexity of the sample. Straightforward genomic DNA sequencing is not economically feasible for individual groups due to the large genome size of tetraploid (~11,000 Mb) and hexaploid (~16,000 Mb) wheat even with the current sequencing capabilities. Therefore, alternative approaches must be used. Genome capture [37] is currently being pursued by several groups working on polyploid wheat [38], although this approach is inherently limited to the genes that are defined on the capture array. As discussed above, the current wheat unigene set is probably not complete enough for the precision that is needed in mapping projects. An alternative method is RNAseq [39], representing an open platform that can detect novel transcripts as long as they are expressed to levels compatible with the achieved sequencing depth.

In this study, we used RNAseq as the strategy for complexity reduction in tetraploid wheat. This method has been successfully implemented for SNP discovery in several species and focuses on just a fraction of the complete genome. For example, the NCBI reference has a gene space of 31,671,110 bp per genome equivalent, accounting for less than 1% of the complete tetraploid genome. We also consciously used non-normalized RNA samples as the downstream BSA requires quantitative estimates of frequency ratios, which would be perturbed by a normalization procedure. However, this generates a major drawback as 50% of all mapped reads correspond to just a minute fraction of very highly expressed unigenes (< 1% in LDN and < 2.5% in RSL65). After accounting for these 'lost' reads and those not mapping to the NCBI reference, less than 30% of all raw reads generated (~15 million reads) were used for the vast majority of the SNP discovery and BSA analysis. This though still provides an average coverage of 23-fold per genome for each unigene.

The use of RNAseq for genome reduction can also generate discrepancies between SNP calling in RNA samples and SNP validation in genomic DNA (gDNA). Expression differences between homoeologous genomes led to SNPs being identified in the RNAseq data, but these putative SNPs were not identified subsequently in the DNA samples. This confounded our results and was further accentuated by the low coverage used in the SNP detection (discussed below, Additional File 1: Table S1). In addition, the more variable nature of RNA expression data compared to gDNA meant that BFR values were not perfectly correlated with their map position across *GPC-B1 *(Additional File 1: Figure S3). Despite these apparent inefficiencies, our results suggest that non-normalized RNAseq was a successful strategy for both SNP discovery and BSA analysis in tetraploid wheat.

### SNP discovery and validation

The initial mapping of reads was conducted with the Maq default parameter of 70 for maximum summed quality scores of mismatched bases. In polyploid species, a high quality mismatch is given not only by varietal SNPs, but also by the homoeologous SNPs between genomes. Since homoeologous wheat transcripts are approximately 97% identical [27], we relaxed the mapping criteria to a value of 120 to allow for 3 high quality IHPs across the reads plus an additional varietal SNP (Maq-120 analysis). This better reflects the biology and the fact that the unigene reference is based on wheat ESTs, which represent all three homoeologues and have been collapsed into a single consensus sequence.

As expected, the Maq-120 parameters increased the percentage of mapped reads for all samples and outperformed Maq-default by identifying 29% more putative SNPs across 22% more unigenes. The validation rate of putative SNPs was very similar between methods (58.5% and 56.1% for Maq-default and Maq-120, respectively). The Maq-120 analysis provided the ability to detect varietal SNPs close to IHPs (Additional File 1: Figure S5) that were missed originally when using the Maq-default parameters. However, by relaxing the overall mapping parameters, several confirmed varietal SNPs from the Maq-default analysis were discarded because the corresponding unigenes had SNP densities above 5 SNPs/kb. This highlights the difficulties in finding the right balance in the mapping parameters and exemplifies potential drawbacks of each approach.

The ability to detect rare transcripts is an important aspect of the SNP discovery and BSA strategy, especially in polyploid species with hemi-SNPs. The Lander-Waterman Model [40] provides an initial estimation of 8-fold coverage to randomly sample a read with 99.97% probability, but this does not account for the tetraploid nature of the sequences examined in this study and assumes that reads will be randomly sampled. Wendl and Wilson [41] address the issue of coverage in heterozygous samples from diploid organisms, which serves as an approximation to our study. Based on their calculations, the probability that at least two independent reads of both parental alleles will be sampled with 16-fold coverage is 99.39%, whereas with only 8-fold coverage this probability drops to 82.53% (for one read, these values are 99.93% and 96.37%, respectively). The fact that we analyzed expressed sequences adds an extra layer of variation, although the high correlation in expression of parental alleles (R^2 ^= 0.85) suggests a relative balance between the lines.

**Figure 7:**
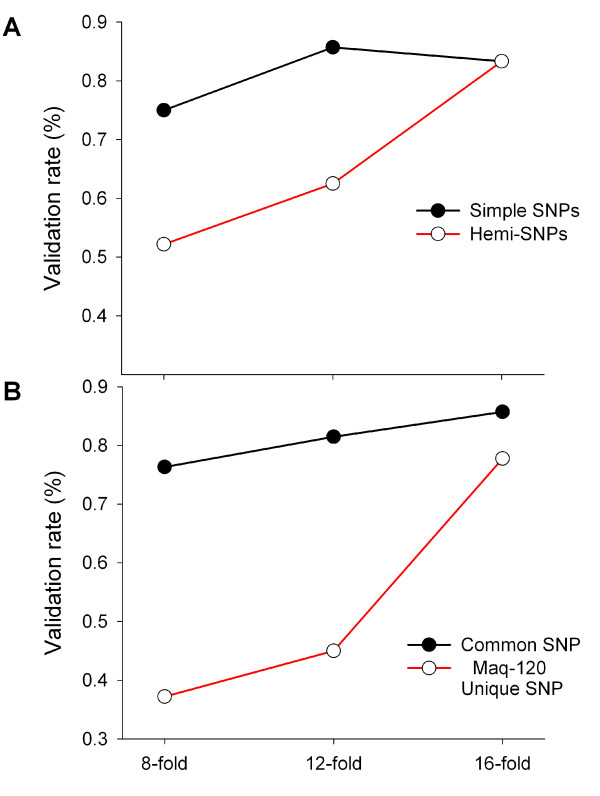
**Validation rates of Maq-120 SNPs according to SNP features**. **A**) SNPs classified as hemi- (red line) or simple-SNPs (black line). **B**) Common SNPs are those identified in both the Maq-default and Maq-120 analyses (black line), whereas the Maq-120 unique SNPs (red line) where only identified in Maq-120. The total number of SNPs evaluated at each coverage depth where 81 (8-fold), 47 (12-fold), and 30 (16-fold).

Our results confirmed that the 8-fold coverage was not sufficient to account for sampling variation within the bulk samples. The composition of the examined Maq-120 SNPs suggests that hemi-SNPs had a lower validation rate than simple SNPs at 8-fold coverage. This is expected from the discussion above, as simple SNPs can be considered as single copy genes that would follow the Lander-Waterman Model more closely. The majority of the putative hemi-SNPs, which were later confirmed to be monomorphic, appeared to be represented by only one homoeologous transcript in the bulk alignment, as determined by the absence of linked IHPs within the read. With increased coverage, the percentage of validated hemi-SNPs increased from 52%-83%, whereas simple SNPs increased marginally from 75%-83% (Figure 7A). A similar analysis on the Maq-120 unique SNPs revealed that validation rates increased to 78% when considering those SNPs identified with at least 16-fold coverage (Figure 7B). A detailed breakdown of SNPs lost during each step, and a summary of the possible reasons, is provided in Additional File 1: Table S1. Taken together, these results suggest that a minimum coverage of 8-fold per diploid genome is an effective way to increase the validation rate by simply decreasing the sampling error.

SNP validation was greatly facilitated by the development of novel genotyping technologies that are flexible and allow a rapid assay design at a relatively low cost. We initially validated SNPs using the SSCP method, as it allows the resolution of polymorphisms in co-amplified homoeologues from a PCR reaction. This circumvents the design of homoeologue specific assays, which are normally required for most genotyping platforms. Although this method proved reliable, it has limitations in that the putative SNP is not directly assayed and that it is an overnight gel-based system that is not high-throughput (roughly 60 samples per gel). Therefore, for the Maq-120 putative SNPs we used the KASPar platform, which is a PCR based system that only requires a three minute end-point fluorescence analysis and which has a higher throughput than SSCP. KASPar allows the direct assay of the putative SNP as the two alternative bases are positioned in the 3' end of the two competing PCR primers (Additional File 1: Figures S5 and S6). Both methods require a preliminary annotation of the sequence as intron positions, which are anonymous in the RNAseq data, must be considered; but this is now a minor step with the publicly available 5× Chinese Spring wheat genome. Importantly, KASPar has proven to be extremely flexible and robust in polyploid wheat and is quickly becoming an important marker system in wheat research [42] and breeding programmes (S. Dreisigacker, CIMMYT, personal communication).

### BSA and mapping

The resolution of the BSA is given by the marker density and by the combined recombinations within each bulk. In wheat, marker densities are variable and depend on the relatedness of the parental lines, marker systems employed, number of individuals examined, and the genome being studied (D genome being the least polymorphic). Regardless of these factors, most genetic maps in wheat have at best an average mapping resolution of 5-10 cM. This includes maps between durum and emmer wheat using a combination of SSR and DArT markers (average 7.5 cM between markers [43]), as well as the recently published map of two UK elite lines that includes SSR, DArT and over 500 KASPar markers (average 4.7 cM between markers [42]). These relatively low marker densities, compared to sequenced genomes, limit the mapping resolution of BSA. We believe that this has now changed with the advent of NGS approaches, which allow an unprecedented number of SNPs to be identified and evaluated in polyploid wheat. This suggests that the resolution of BSA in wheat will be dependent on the approach used for complexity reduction (discussed above) and the recombinations within each sequenced bulk.

The first NGS studies in *Arabidopsis *re-sequenced DNA from large bulks of ~500 F_2 _individuals to map specific EMS mutations [18]. Recent approaches have shown consistent results with 50 F_2 _individuals by exploiting a larger number of SNPs [19]. Both approaches take advantage of the physical map and known gene content in *Arabidopsis*, focus on specific EMS-induced transitions that lead to amino acid changes in proteins, and benefit from the low mutation densities in *Arabidopsis *(~1 EMS mutation per 100-200 kb, or ~1-2 cM) [17]. This means that in *Arabidopsis*, mapping a mutation to within 300-400 kb should suffice as the number of candidate SNPs corresponding to EMS mutations *and *leading to amino acid changes should be very small. In maize, several recessive mutant phenotypes have been mapped using a BSA and quantitative genotyping (Sequenom) approach [44]. In this study, a genome wide scan was conducted using bulks of at least 20 F_2 _individuals, and mapping intervals of several cM were achieved.

In our study, we used bulks composed of a seemingly small number of individuals; 14 and 13 homozygous F_3 _recombinant lines per bulk. These lines originate from a large screen of ~4,500 F_2 _plants and were selected based on the presence of recombination across a 12.2 cM interval to which *GPC-B1 *was previously mapped. Therefore, although the absolute number of individuals is low with respect to other studies, the amount of information in terms of recombination is high. Considering equal probability of recombination for all 27 individuals across the 12.2 cM interval, we expected to achieve a mapping resolution of 0.45 cM.

In wheat, a bespoke physical map is required for each positional cloning project making the ability to rapidly achieve sub-cM intervals critical for success. Based on previous experience, one BAC clone is roughly equivalent to 0.1 cM, therefore small genetic distances (0.3-0.5 cM) are usually required before initiating the physical map to reduce the number of chromosome walking steps. Using a traditional mapping approach with individual marker development based on collinearity, we had previously mapped *GPC-B1 *to a 0.3 cM interval defined by rice genes Os02g04520 and Os02g04630 (Additional File 1: Table S2) [11,14]. In this study, we mapped *GPC-B1 *to a genetic interval of 0.4 cM defined by Os02g04490 and Os02g04650. Although we did not achieve single-BAC resolution, this compares favourably considering that the proximal flanking marker Ta#S17984935 shares homology to Os02g04650, only one gene away from the end of the physical map (Os02g04640) developed for the cloning of *GPC-B1*, and the distal flanking marker Ta#S37941845 is homologous to Os02g04490, just five genes away in rice to the distal end of the physical map, Os02g04550.

This very narrow mapping interval was achieved in a matter of months as opposed to years, although it is important to consider that we benefitted from high quality phenotypic data and germplasm to assemble the bulks. The new genotyping and sequencing technologies will reduce the time and effort required for many steps such as screening for recombinant plants, identifying polymorphic markers, and genotyping the individuals from the bulks. However, the reliable phenotyping of plants will remain a major determinant in the successful outcome of any BSA and mapping project. Having said this, these new technologies now allow for several genes to be targeted in parallel by a single person and for BSA to be conducted in species with no genome sequence information.

### Future directions

Our results suggest that NGS-enabled genetics should be feasible in polyploid species and highlight several areas for further improvement in wheat genomics. An important first step would be to produce a more comprehensive and definitive unigene set for wheat. There are several ongoing efforts which should be coordinated to develop a publicly available gene set for all wheat researchers. We expect a catalogue of IHPs to be available for the three wheat genomes and an initial assignment of SNPs should be possible based on the diploid progenitors. This strategy has been used successfully before [45] and the publicly available *Ae. tauschii *raw sequences [46] are a good starting point for the D genome. The combination of a comprehensive gene set with IHPs will make the varietal SNP detection pipeline much more efficient by initially masking for IHPs and then using these to assign genomes. By linking IHPs to varietal SNPs, it should be possible to automate the design of homoeologue specific primers. Flexible platforms for SNP detection, such as KASPar, will make this approach even more robust and accessible to a large number of research groups. An important final element is the release of sequences for the individual wheat chromosome arms. Currently, sequences are publicly available for group 1 [32] and group 7 [47] chromosomes and the remaining sequences are currently being generated [48]. Combining the information outlined above would result in a comprehensive set of unigenes, with haplotype information for each genome, and mapped to the corresponding chromosome arms. This would represent a step-change in wheat genomics and would significantly enhance the NGS-BSA approach.

## Conclusions

In this study, we outline a method that combines SNP discovery by RNAseq with BSA for fine-mapping genes in polyploid wheat. A balanced set of validated SNPs from both parents was generated and ~70% mapped within the targeted interval. The SNPs were well distributed and allowed the identification of almost all recombination events, successfully mapping a gene (*GPC-B1*) to a 0.4 cM interval. The large number of SNPs also generated a high density haplotype across the region, which in future projects will be useful for breeding purposes.

## Methods

### Plant material and growing conditions

The parental lines used in this study were tetraploid wheat cultivar 'Langdon' (LDN) and a recombinant substitution line (RSL65) from the cross LDN (DIC-6B) × LDN [4]. The homozygous recombinant lines used for the bulks and fine-mapping were either sister lines of RSL65 (generated from the same cross and with identification numbers less than 100) or were generated from the cross LDN × RSL65 (with identification numbers greater than 100). Their genotypes and grain protein concentration phenotypes have been described before [4,5,9,14]. Briefly, 14 RSLs with known high protein (8, 28, 50, 54, 58, 65, 117, 121, 147, 152, 158, 241, 259, 300) and 14 lines with low protein (14, 19, 59, 77, 78, 129, 135, 148, 209, 289, 290, 293, 215, 3417) phenotype were used. These lines carry recombination events in homozygous state across the ~12-cM interval, which includes *GPC-B1 *and is delimited by markers *Xwms508 *and *Xwms193*.

To minimize differences in growth conditions between plants with an opposite phenotype, pairs of high and low protein RSLs were grown together in 2 L pots and properly labelled. Four biological replicates of each high-low pairing were grown, but only one pot was selected for sampling based on visual comparison between the high and low RSLs. The top third of the 5^th ^leaf was collected for DNA extraction, whereas the bottom third was collected for RNA extraction (the middle third was kept as back-up).

### Preparation of samples and RNA bulks

DNA from individual samples was prepared as described previously [49] and analyzed for their genotype across the *GPC-B1 *interval using markers *Xuhw89 *(distal), *Xucw71 *(proximal) and *Xucw101 *(causal SNP at *GPC-B1*) using published conditions [11,14]. Total RNA was prepared by grinding the bottom third of the 5^th ^leaf in liquid nitrogen and extracting RNA using TRIzol (Invitrogen) according to the manufacturer's protocol. RNA concentration was measured using 1 μL of each RNA sample on the NanoDrop ND-1000 Spectrophotometer. RNA quality was assessed by running 1 μL of each RNA sample on an Agilent RNA 6000 n LabChip (Agilent Technology 2100 Bioanalyzer). Samples with an RNA Integrity Number (RIN) value greater than eight were deemed acceptable according to the Illumina mRNA-Seq protocol. Equal amounts of RNA from the 14 individuals previously classified as high protein were mixed to produce the high protein RNA bulk. The low protein RNA bulk was constructed using the RSLs described above except for RSL 135, which was found to be heterozygous in the DNA marker analysis and therefore excluded. To maintain a balanced set of alleles at the flanking loci, we added double the amount of RNA from RSLs 77 and 78 to the low protein bulk, which therefore included RNA from 15 RSLs (13 distinct genotypes).

### Illumina library production

The Illumina mRNA-Seq 8-Sample kit (RS-100-0801, Illumina Inc.) was used according to the manufacturer's protocol with the following modifications. In brief, poly-A containing mRNA molecules were purified from 5 ug total RNA using poly-T oligo attached magnetic beads. The purified mRNA was fragmented by addition of 5× fragmentation buffer (Illumina, Hayward, CA) and was heated at 94°C in a thermocycler with 2 different times (2 min and 5 min). The fragmentation time of 5 min is the standard time used in the protocol, which yields fragments of ~250 bp. The shorter fragmentation time was used to yield slightly larger library fragments of 350-400 bp. First strand cDNA was synthesised using random primers to eliminate the general bias towards 3' end of the transcript. Second strand cDNA synthesis was done by adding GEX second strand buffer (Illumina, Hayward, CA), dNTPs, RNaseH and DNA polymerase I followed by incubation for 2.5 h at 16°C. Second strand cDNA was further subjected to end repair, A-tailing, and adapter ligation in accordance with the manufacturer supplied protocols. Purified cDNA templates were enriched by 15 cycles of PCR for 10 s at 98°C, 30 s at 65°C, and 30 s at 72°C using PE1.0 and PE2.0 primers and with Phusion DNA polymerase (Illumina, Hayward, CA). The samples were cleaned using QIAquick PCR purification columns and eluted in 30 μl EB (Elution Buffer) as per manufacturer's instructions (QIAGEN, CA). Purified cDNA libraries were quantified using Bioanalyzer DNA 100 Chip (Agilent Technology 2100 Bioanalyzer).

### Illumina library clustering and sequencing conditions

Parental libraries were normalized to 7.5 nM in EB (Qiagen). Samples were then diluted to 1.5 nM with NaOH (4 μL of 10 nM stock, 1 μL of 2 N NaOH and 15 μL EB) and left at room temperature for 2 min before transferring 4 μL into 496 μL of HT1 (High salt buffer supplied with cluster kit Paired-End Cluster Generation Kit V4 PE-203-4001, Illumina) to give a final concentration of 12 pM. Each bulk library was normalised to 10 nM in EB, diluted to 2 nM with NaOH and 2.5 μL transferred into 497.5 μL HT1 to give a final concentration of 10 pM. 120 μL of normalised library was then transferred into a 200 μL strip tube and placed on ice before loading onto the Cluster Station, each library being run on a single lane. Flow cells were clustered using Paired-End Cluster Generation Kit V4, following the Illumina PE_amplification_Linearization_Blocking_PrimerHyb_v7 recipe. Following the clustering procedure, the flow cell was loaded onto the Illumina Genome Analyzer GAIIx instrument following the manufacturer's instructions. The sequencing chemistry used was v4 (FC-104-4001, Illumina) using software SCS 2.6 and RTA 1.6. Each parental library was run in a single lane for 120 cycles for each paired end, and each bulk library for 80 cycles. Illumina base calling files were processed using the GERALD pipeline to produce paired sequence files containing reads for each sample in Illumina FASTQ format.

### Computational methods

After first converting the Illumina FASTQ files to Sanger FASTQ format, initial alignment of paired reads from single lanes was conducted using Maq v0.7.1 [50] against a wheat transcriptome reference comprising 40,349 unigene sequences totalling 31,671,110 bases (NCBI TA build 57) [25]. In the first experiments Maq default parameters were used. In subsequent experiments (referred to as Maq-120 in the text) the maximum summed quality score of mismatched bases was set to 120 (default 70) at each step in the workflow. This allowed SNP haplotypes occurring within the 120 base reads to align with higher mapping quality. Maq-120 alignment was also performed on the component of reads that failed to map to the NCBI Unigene reference against a new reference constructed from a 5× assembly of wheat Chinese Spring genomic 454 reads (M. Bevan, JIC, personal communication). For the bulk samples, maps constructed separately from the two library size fractions were merged before further processing. SNPs were detected and scored by methods previously developed for the polyploid oilseed rape *Brassica napus *[21,28]. Crucially, it was expected that reads originating from homoeologous genes would be mapped to the same unigene reference. Briefly, Maq was used to call SNPs with respect to the reference for each parental line separately and then the SNP_parser.pl Perl script (Additional File 5) was used to derive the symmetric difference (A Δ B) of the two sets. Base calls at SNP positions together with quality scores were then programmatically compared and re-assessed by accessing the verbose pileup files generated from the Maq alignments, thus producing a filtered set of SNPs between the parents. This was done at different minimum depth thresholds (8-fold, 12-fold, and 16-fold). The Illumina reads for parental and bulk samples were deposited in the EMBL-EBI Sequence Read Archive (ERA050658).

A new Perl script bulk_frequencies.pl (Additional File 6) was developed to analyse allele frequencies for the parental SNPs between the two bulks. This used an indexing method [28] for fast access to individual lines in the pileup files in order to extract base calls and quality scores for each SNP position. For every hemi-SNP, for instance Y (i.e. C/T) from parent LDN versus C from parent RSL65, the frequency of the informative base (in this case T) was calculated for each bulk and then the ratio of this frequency between the bulks (BFR) was determined. The expectation was that a hemi-SNP coinciding with the trait/gene should be revealed by informative base frequencies tending to either 0.5 or to zero in the two bulks, depending on the parental origin. SNPs were filtered using a BFR of ≥ 3.0 as a threshold, after excluding divide-by-zero errors. Simple SNPs were processed in a similar fashion.

Unigenes that showed apparent SNP densities of greater than 5 SNPs/kb were considered artefactual (or paralogous) and excluded from the analysis (the SNP density between LDN and RSL65 had been experimentally determined as 2.2 SNPs/kb). The BFR results were organized in a spreadsheet format to aid further inspection and sorting. Ancillary synteny data for each unigene was added, including the best hit for the unigene from pre-computed BLASTN analysis against *Brachypodium*, rice and sorghum gene models (E-value cut-off 1E-50), together with a measure of transcript abundance, expressed as reads per kilobase per million mapped reads (RPKM values).

### Marker design and SNP assays

To design markers targeting the putative SNPs, the 250-bp surrounding the candidate SNP on either side were extracted from the unigene. These sequences were annotated for exon-intron positions using BLASTN analysis against the 5× genomic sequence of wheat cultivar Chinese Spring (454 raw reads, unassembled) [30]. Sequences containing putative SNPs with over 100 hits at 1E-50 were considered repetitive and were not processed further. Primers for the SNPs identified in the Maq-default analysis were designed to amplify ~150-200 bp fragments as the initial screens were based on single strand conformation polymorphism (SSCP) of PCR products. The second set of SNPs from the Maq-120 analysis was annotated using a similar approach, but primers were designed to amplify products for KASPar assays [42,51] when possible. PCR conditions and SSCP analysis were done using published protocols [49]. KASPar oligos were ordered from Sigma-Aldrich, with primers carrying standard FAM or VIC compatible tails (FAM tail: 5' GAAGGTGACCAAGTTCATGCT 3'; VIC tail: 5' GAAGGTCGGAGTCAACGGATT 3') and the target SNP in the 3' end. Primer mix was set up as recommended by Kbioscience (46 μl dH_2_O, 30 μl common primer (100 μM), and 12 μl of each tailed primer (100 μM)) [51]. Assays were tested in 384-well format and set up as 5 μl reactions (2.5 μl template [10-20 ng of DNA], 2.43 μl of V3 2xKaspar mix, and 0.07 μl primer mix). PCR was performed on a Peltier PTC-225 PCR tetrad machine fitted retrospectively with 384 blocks using the following protocol: Hotstart at 95°C for 15 min, followed by ten touchdown cycles (95°C for 20 s; Touchdown 65°C, -1°C per cycle, 25 s) and then followed by 26 cycles of amplification (95°C 10 s; 57°C 60 s). Since KASPar amplicons are usually smaller than 120 bp, no extension step is necessary in the PCR protocol. 384-well sample plates (Cat. No. 04729749001, Roche Diagnostics) were read on a Roche Lightcycler^® ^II 480 qPCR machine. Fluorescence was detected at ambient temperature (20-25°C; RAMP speed 0.05°C per s) with four detection steps per °C. If the signature genotyping groups had not formed after the initial amplification, additional amplification cycles (usually 5-10) were applied, and the samples were read again. Data analysis was performed manually using the inbuilt Roche Lightcycler^® ^480 software (Version 1.50.39). A full list of primers is provided (Additional File 7).

### Accession codes

Short read sequence data reported here have been deposited at the Sequence Read Archive (SRA) under the accession code ERA050658.

## Abbreviations

BAC: Bacterial artificial chromosome; BFR: Bulk frequency ratio; BSA: Bulked segregant analysis; cM: centi-Morgan; DArT: Diversity array technologies; DIC6B: *Triticum turgidum *ssp. *dicoccoides *chromosome 6B substitution line; DNA: Deoxyribonucleic acid; EST: Expressed sequence tag; gDNA: Genomic DNA; *GPC-B1: *Grain protein content gene; IHP: Inter-homoeologue polymorphisms; KASPar: KBiosciences competitive allele specific PCR genotyping system; LDN: Langdon; NCBI: National Center for Biotechnology Information; NGS; Next-generation sequencing; PCR: Polymerase chain reaction; RNA: Ribonucleic acid; RNAseq: mRNA sequencing; RPKM: Reads per kilobase per million mapped reads; RSL: Recombinant substitution line; SHOREmap: Short read mapper; SNP: Single nucleotide polymorphism; SSCP: Single strand conformation polymorphism; SSR: Simple sequence repeat.

## Authors' contributions

MT and CU conceived and designed the experiments; MF performed library construction, NMA, SGM, and CCJ carried out the SNP validation and mapping experiments; MT and CU performed data analysis; MT performed bioinformatic analysis; MT, NMA, CCJ, and CU drafted and revised the manuscript. All authors read and approved the final version of the manuscript.

## Supplementary Material

Additional file 1 **Figure S1: **Flow-chart summarizing the main steps of the NGS-BSA approach. **Figure S2: **Chromatograms of LDN and RSL65 for Ta#S32574498. **Figure S3: **BFR of validated and mapped SNPs across the *GPC-B1 *interval. **Figure S4: **Mapping of wheat unigenes with putative SNP to the *Brachypodium *genome. **Figure S5: **View of MAQ alignment of Ta#S37941845 reads for LDN and RSL65 encompassing the hemi-SNP (R = A/G) at position 582. **Figure S6: **Visualization of fluorescence output for two hemi-SNPs **Table S1: **Breakdown of SNPs lost during the validation and mapping process **Table S2: **Collinearity between most closely identified markers in the present study and previous markers used for physical map construction. Click here for file

Additional file 2 **Characteristics of all putative SNPs identified in the bulk samples by Maq-default analysis**. Click here for file

Additional file 3 **Characteristics of all putative SNPs identified in the bulk samples by Maq-120 analysis**. Click here for file

Additional file 4 **Characteristics of SNPs mapped to the *GPC-B1 *region**. Click here for file

Additional file 5 **SNP_parser.pl Perl script**. Click here for file

Additional file 6 **bulk_frequencies.pl Perl script**. Click here for file

Additional file 7 **Primers used in this study and characteristics of all SNPs tested**. Click here for file
