# Supplementary material for: Combining SNP discovery from next-generation sequencing data with bulked segregant analysis (BSA) to fine-map genes in polyploid wheat
Source: BMC Plant Biol. 2012 Jan 26;12:14. doi: 10.1186/1471-2229-12-14 (PMC3296661; doi:10.1186/1471-2229-12-14)
Supplement: Additional file 1 — Figure S1: Flow-chart summarizing the main steps of the NGS-BSA approach. Figure S2: Chromatograms of LDN and RSL65 for Ta#S32574498. Figure S3: BFR of validated and mapped SNPs across the GPC-B1 interval. Figure S4: Mapping of wheat unigenes with putative SNP to the Brachypodium genome. Figure S5: View of MAQ alignment of Ta#S37941845 reads for LDN and RSL65 encompassing the hemi-SNP (R = A/G) at position 582. Figure S6: Visualization of fluorescence output for two hemi-SNPs Table S1: Breakdown of SNPs lost during the validation and mapping process Table S2: Collinearity between most closely identified markers in the present study and previous markers used for physical map construction. [file 1471-2229-12-14-S1.PDF]

Additional File 1

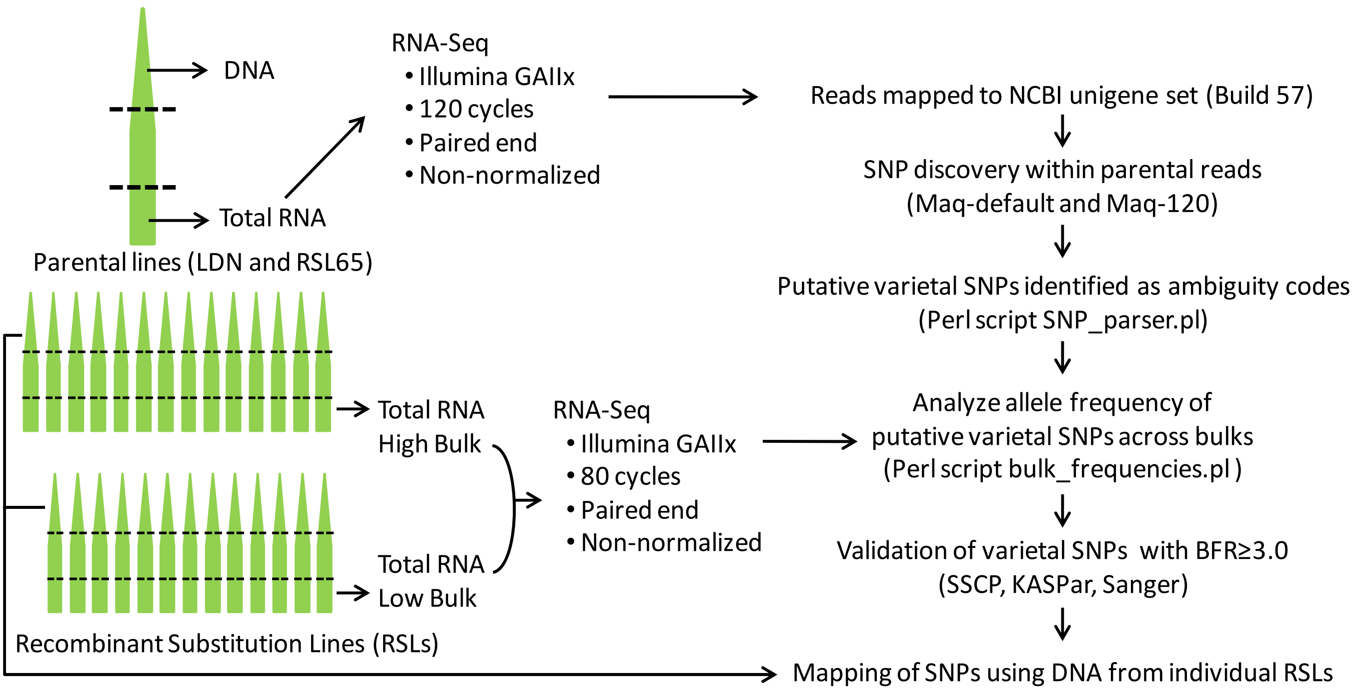

Figure S1. Flow-chart summarizing the main steps of the NGS-BSA approach.

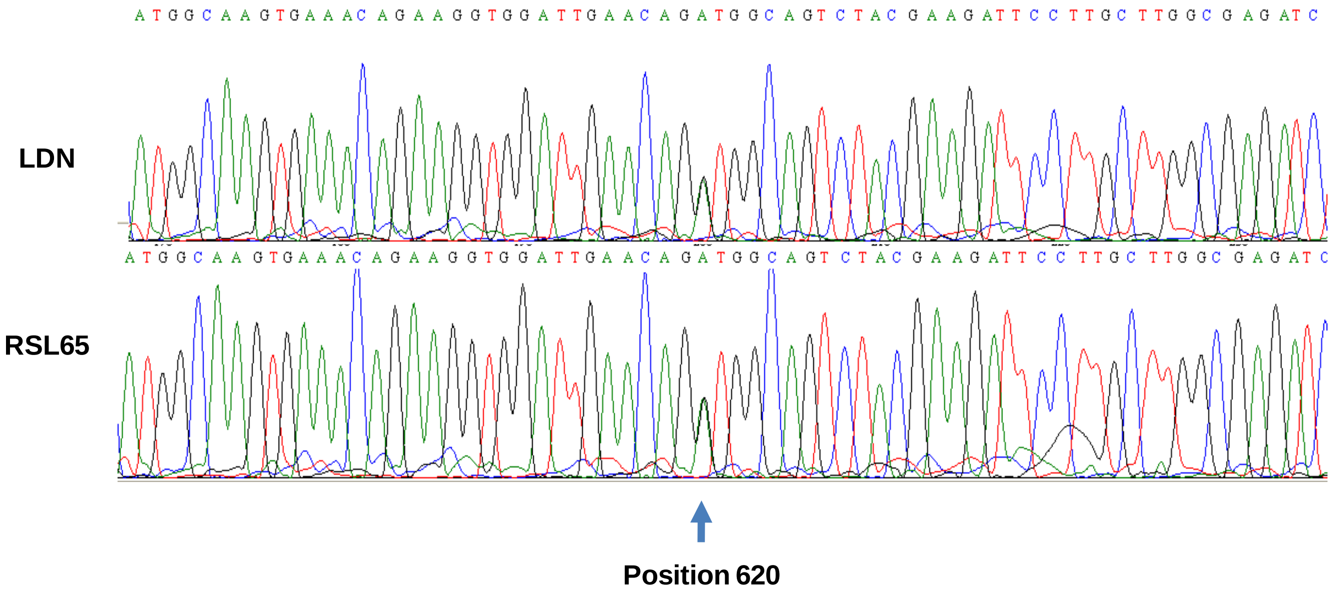

Figure S2. Chromatograms of LDN (top) and RSL65 (bottom) for Ta#S32574498. Both gDNA traces show the presence of an IHP (R=A/G) at position 620, confirming the monomorphic nature of this putative hemi-SNP (R620A) in the parental lines.

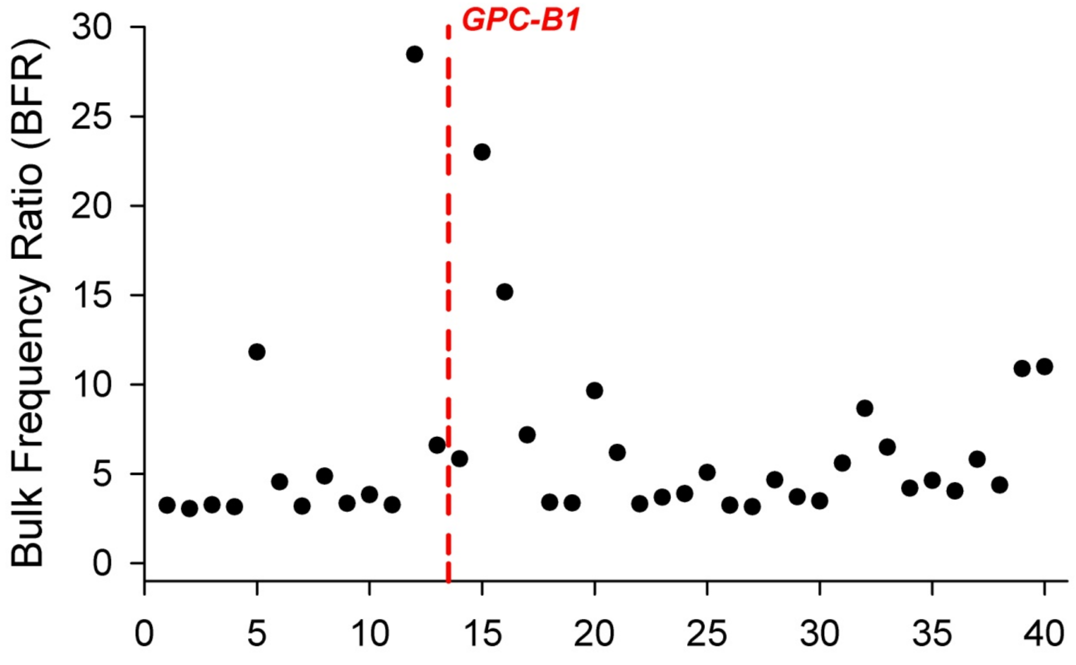

Figure S3: BFR of validated and mapped SNPs across the *GPC-B1* interval. SNP markers are plotted along the X-axis according to their map position. The broken red line indicates the position of the *GPC-B1* gene.

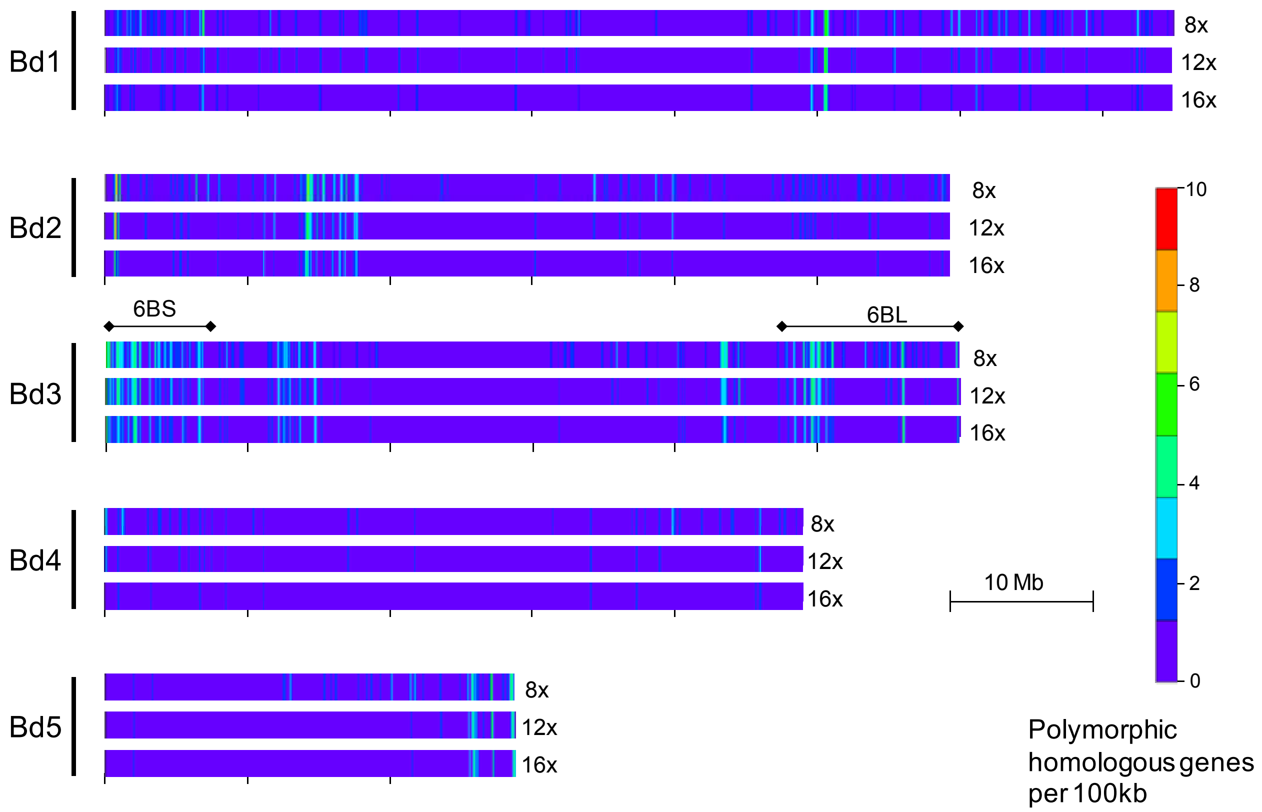

Figure S4: Mapping of wheat unigenes with putative SNPs to the *Brachypodium* genome. Each *Brachypodium* chromosome is coloured according to the density of polymorphic homologous genes per 100 kb identified by the Maq-120 analysis on the parental LDN and RSL65 lines. Three different coverage depths (8x, 12x, 16x) are represented and the collinear regions to wheat chromosome arms 6BS and 6BL are indicated.

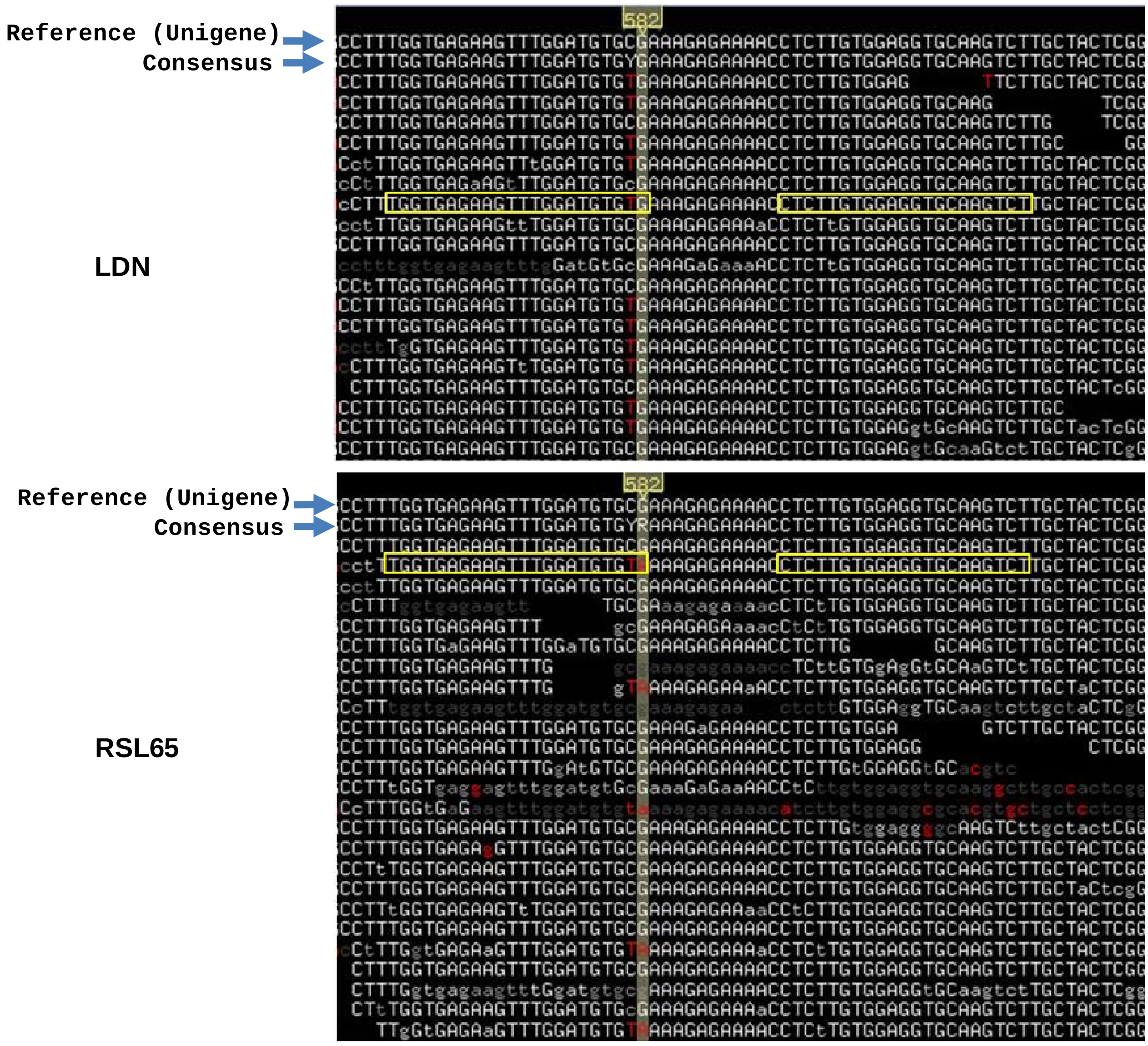

Figure S5. View of MAQ alignment of Ta#S37941845 reads for LDN (top) and RSL65 (bottom) encompassing the hemi-SNP (R=A/G) at position 582. The presence of the IHP (Y=C/T) at position 581 allows the design of homoeologue-specific primers based on the linkage of the T at position 581 with the A hemi-SNP at position 582. Yellow boxes correspond to the primer sequence of the allele specific (forward) and the non-specific (reverse) primer. The top sequence in the alignments corresponds to the NCBI Unigene reference, the second to the consensus, and the raw reads follow below. Red letters correspond to base changes with respect to the reference and grey lowercase letters correspond to positions with low quality score (Q<20).

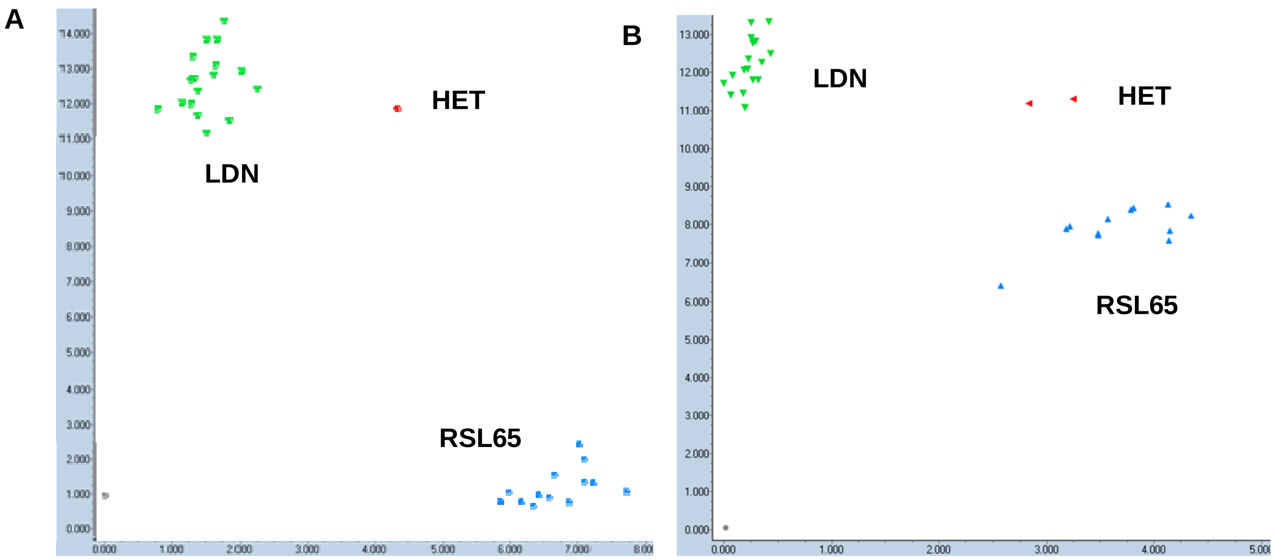

Figure S6. Visualization of fluorescence output for two hemi-SNPs. **A)** KASPar output for Ta#S37941845, which is a homoeologue-specific assay (Fig S2) and, **B)** Ta#S13117987, which is a non-specific assay. Coloured triangles represent genotype of homozygous RSLs for LDN (green) or RSL65 (blue) genotypes. A heterozygous control is shown in red, whereas the non template control is shown by a grey circle near the origin of the graph. The additional heterozygous sample in panel B corresponds to RSL 290 which was heterozygous for additional markers in the region.

Table S1. Breakdown of SNPs lost during the validation and mapping process. SNPs were categorized as originating from the Maq-default and Maq-120 analysis. The loss of SNPs at each step is not due to a particular mapping or SNP calling criterion.

**Technical Step**	**SNPs**[1]	**Effic.**[2]	**Possible reasons for lost SNPs**
Putative varietal SNPs identified across both bulks			
Maq-default	1,619 of 2,427	66.7%	Insufficient coverage in both bulks (minimum of 8x)
Maq-120	3,172 of 4,430	71.6%	SNP miscalled in parental lines (i.e. putative SNP not identified in bulks)
Varietal SNPs (BFR≥3.0) validated in parental lines			
Maq-default	48 of 82	58.5%	Low coverage leads to high false-positive rate (especially for hemi-SNPs)
Maq-120	46 of 82	56.1%	Expression differences between homoeologous genomes leads to SNP in RNA data, but not at the DNA level.
SNPs mapping to *GPC-B1* interval			
Maq-default (8x)	31 of 48	64.6%	Segregation of an additional wild emmer segment in a subset of RILs.
Maq-120 (8x)	31 of 46	67.4%	*Trans*-acting expression effect leads to difference in RNA profile between bulks, but affected gene maps outside *GPC-B1* interval.

[1] = Refers to number of SNPs which satisfy the criteria indicated in the technical step.
[2] = Efficiency of each technical step with regards to SNPs which satisfy the criteria.

Table S2. Collinearity between the most closely identified markers in the present study and previous markers used for the physical map construction. Lack of collinearity is indicated by three dashes.

Marker / Unigene	*Brachypodium*	Rice	Sorghum	Comment
Ta#S16259088	Bradi3g03340	Os02g04500	Sb04g003030	
Ta#S37941845	Bradi3g03330	Os02g04490	Sb04g003020	Distal flanking marker identified by BSA
Xucw79	Bradi3g03350	Os02g04520	Sb04g003060	
Xucw83	Bradi3g03390	Os02g04550	Sb04g003100	Distal end of physical map
GPC-B1	---	---	---	
Xucw84	Bradi3g03550	Os02g04560	Sb04g003130	
Xucw71	---	Os02g04630	---	
Xucw96	Bradi3g03540	Os02g04640	Sb04g003140	Proximal end physical map
Ta#S17984935	---	Os02g04650	Sb04g003150	Proximal flanking marker identified by BSA
Ta#S32700697	Bradi3g03530	Os02g04660	Sb04g003160	
